# Role of Mast Cells in Shaping the Tumor Microenvironment

**DOI:** 10.1007/s12016-019-08753-w

**Published:** 2019-06-29

**Authors:** Daniel Elieh Ali Komi, Frank A. Redegeld

**Affiliations:** 1 Immunology Research Center, Tabriz University of Medical Sciences, Tabriz, Iran; 2 Department of Immunology, Tabriz University of Medical Sciences, Tabriz, Iran; 3 Division of Pharmacology, Department of Pharmaceutical Sciences, Faculty of Science, Utrecht University, Universiteitsweg 99, 3584CG Utrecht, The Netherlands

**Keywords:** Cancer, Extracellular matrix, Immunosuppression, Mast cell, Tumor

## Abstract

Early mast cell (MC) infiltration has been reported in a wide range of human and animal tumors particularly malignant melanoma and breast and colorectal cancer. The consequences of their presence in the tumor microenvironment (TME) or at their margins still remain unclear as it is associated with a good or poor prognosis based on the type and anatomical site of the tumor. Within the tumor, MC interactions occur with infiltrated immune cells, tumor cells, and extracellular matrix (ECM) through direct cell-to-cell interactions or release of a broad range of mediators capable of remodeling the TME. MCs actively contribute to angiogenesis and induce neovascularization by releasing the classical proangiogenic factors including VEGF, FGF-2, PDGF, and IL-6, and nonclassical proangiogenic factors mainly proteases including tryptase and chymase. MCs support tumor invasiveness by releasing a broad range of matrix metalloproteinases (MMPs). MC presence within the tumor gained additional significance when it was assumed that controlling its activation by tyrosine kinase inhibitors (imatinib and masitinib) and tryptase inhibitors (gabexate and nafamostat mesylate) or controlling their interactions with other cell types may have therapeutic benefit.

## Introduction

In addition to tumor cells, a variety of cells (such as stromal cells and fibroblasts), extracellular matrix (ECM), a complicated network of blood-supplying vessels, and molecules (including signaling molecules) together shape the tumor microenvironment (TME) [1]. The TME could be depicted as a smoldering site of inflammation where a large number of infiltrated or resident cells produce and release cytokines, chemokines, and enzymes such as TNF-α, MMP-9, Cox-2, IL-6, iNOS, and VEGF, capable of mediating the inflammatory responses [2]. Maintenance, growth, metastasis, or eradication of tumors depends strongly on external signals received from surrounding immune and non-immune cells of TME [1]. The final consequence of such orchestration of the immune response may be the malignant progression in the TME [2]. The abnormal vasculature system of a tumor cannot sufficiently meet the oxygen requirement of the tumor cells. In return, hypoxic cancer cells release angiogenesis-inducing factors, mainly vascular endothelial growth factor A (VEGF-A), which engages VEGFR2 expressed by endothelial cells (ECs) [3]. MCs localize at the margins of tumors and the TME, commonly around the vessels [4]. The presence of MCs in the tumor structure is not a new finding as Paul Ehrlich already described them in his doctoral thesis in 1878 [5, 6]. MCs are FcεRI+/CD117+ innate immune cells that differentiate from bone marrow–residing hematopoietic progenitor cells [7]. To complete their cycle, the progenitors circulate in the blood to reach target organs by a well-organized trafficking induced by chemoattraction of mediators released from each organ [8]. In addition to stem cell factor (SCF)—the main mast cell (MC) survival cytokine—CXCL12, IL-3, IL-4, IL-9, IL-10, IL-33, and TGF-β are other modulators of survival and growth of MCs [9]. Although most of our knowledge in MC biology is obtained from studying their role in allergic events, a new picture of them as a source of proinflammatory and angiogenic mediators within the tumor has emerged [5] (Table 1). Within the TME, MCs possess both pro- and antitumorigenic properties. Upon activation and degranulation, they become highly proinflammatory and actively recruit cells of the innate immune system mainly neutrophils, macrophages, and eosinophils and cells of the acquired immune system (B and T cells) to orchestrate antitumor immune responses [10]. Conversely, the outcome of their presence could be in favor of tumor progression through releasing VEGF to support angiogenesis and MMP9 to degrade ECM and facilitate the metastasis [10]. The inconsistent and conflicting prognostic value of MC presence in TME may stem in the heterogeneous nature of investigated tumors and animal models [11, 12].

**Table 1:** Previous human studies aimed to determine the role of MCs in shaping TME

Type/site of the tumor	Comments	Ref
Non-small cell lung cancer (NSCLC)	MCs were accumulated in tumors, and both MC_T_ and MC_TC_ were abundant in tumors of patients with extended survival.	[13]
Hodgkin’s lymphoma	Higher rates of MC infiltration in tumors were related to a worse relapse-free survival of patients.	[14]
Colorectal cancer	Infiltration of tryptase-positive MCs is an oncogenic event in colorectal cancer with poor prognosis. Tryptase activates PAR-2 receptor which activation promotes the progression of colorectal cancer.	[15]
Oral squamous cell carcinoma (OSCC)	A significantly higher MC density was observed in lesions compared with control. The presence of MCs in tumors was associated with a better prognosis.	[16]
Breast cancer	The number of tryptase+ MCs in tumors was significantly higher than that of peritumoral and non-tumoral controls	[17, 18]
Prostate cancer	Intratumoral MCs were found protective against prostate cancer recurrence.	[10]
	CD117^+^ MCs showed a denser accumulation in prostate adenocarcinoma (PCa) in comparison with benign prostate tissues that were correlated with the levels of serum prostate-specific antigen (sPSA) and the tumor progression and aggressiveness.	[19]
Cutaneous T cell lymphomas (CTCL)	Infiltration and accumulation of MCs were observed in different rates around CTCL. They accumulate mostly in the area immediately around the tumor.	[20]
Clear-cell renal cell carcinoma (ccRCC)	Infiltrated MC density was negatively correlated with the size of the tumor and reported as a predictor of cancer-specific survival and relapse-free survival in nonmetastatic ccRCC.	[11]
Gastric cancer (GC)	MC density was increased in well-differentiated GC.	[21]
	Tryptase-positive MCs have a role in angiogenesis in the primary tumor and in LNs of patients with metastatic GC.	[22]
Endometrial carcinoma	An increased number of MCs were observed in different stages in which grade III showed the highest MC accumulation. Tryptase-positive MC accumulation was in correlation with angiogenesis and tumor progression.	[23]
Renal cell carcinoma (RCC)	MC infiltration was correlated with angiogenesis and the progression of tumors	[24, 25]
Pancreatic cancer	Increased level of MC-released tryptase in plasma and TME correlates with tumor angiogenesis.	[26]
Thyroid carcinoma	MCD was significantly increased in tumors. Higher rate of MC infiltration was correlated with extrathyroidal extension	[27]
Renal cancer	An inverse correlation was found between the count of accumulated MCs in the peritumorous region and 5-year postoperative patient survival. MCD had a correlation with the tumor size and angiogenesis within peritumorous zone.	[28]

**Fig. 1:**
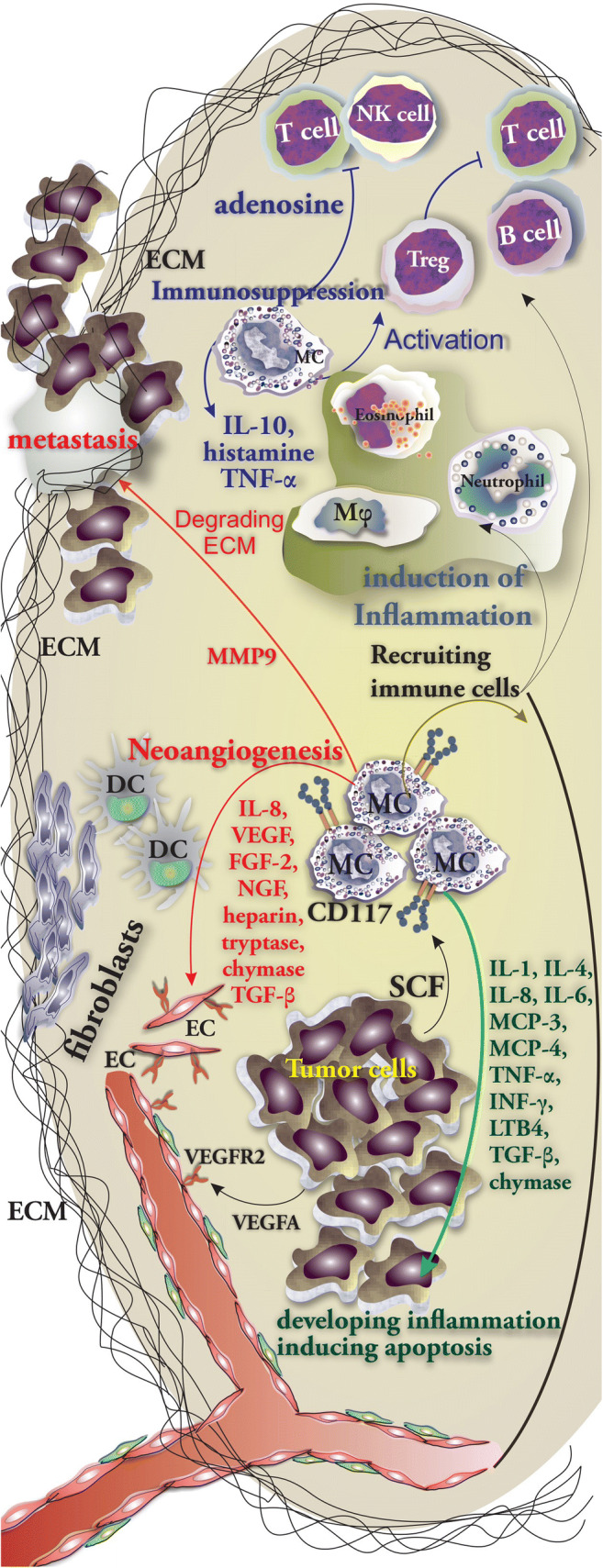
MC orchestration of immune responses in tumor. MC involvement and the role of their mediators in immunity against tumor cells. MCs are able to release a wide variety of cytokines including IL-1, IL-4, IL-8, IL-6, MCP-3, MCP-4, TNF-α, IFN-γ, LTB4, TGF-β, and chymase which support and promote the inflammation, inhibit tumor cell growth, and induce the apoptosis of tumor cell (in green). MC-released mediators mainly IL-8, VEGF, FGF-2, NGF, heparin, tryptase, chymase, and TGF-β support neoangiogenesis (in red). Furthermore, IL-10, histamine, TNF-α, and adenosine possess immunosuppressive properties (in blue)

MCs accumulate into tumor microenvironment by the help of tumor cell-released chemoattractants such as SCF or CCL15 [29]. Within the tumor, MCs release angiogenic compounds including IL-8, VEGF, FGF-2, NGF, heparin, tryptase, chymase, and TGF-β. Additionally, MMP-2 and MMP-9 released by MCs are capable of facilitating the tumor vascularization and promoting tumor invasiveness, respectively [5]. A variety of cytokines released by MCs including IL-1, IL-4, IL-8, IL-6, MCP-3, MCP-4, TNF-α, IFN-γ, LTB4, TGF-β, and chymase contribute to developing inflammation, inhibiting tumor cell growth, and inducing tumor cell apoptosis [5]. In some settings depending on the type of tumor, MCs can have an immunosuppressive role by releasing IL-10, histamine, and TNF-α. Additionally, MCs may suppress T cells and NK cells by releasing adenosine into the microenvironment [2]. Upon infiltration of MCs into the tumor stroma, the expansion and activation of Tregs are promoted. Consequently, Tregs stimulate immune tolerance leading to tumor progression [5] (Fig. 1). Our current knowledge regarding the role of MCs in shaping the TME has been obtained from analyzing the cytology of both animal and human tumors (in vivo) and also co-culturing tumor cell lines with primary MC and MC cell lines (mainly HMC-1 and LAD2) in vitro (Table 2). A great number of mast cell mediators can influence the TME by stimulating angiogenesis, inducing the breakdown of the extracellular matrix, and stimulating tumor growth (Table 3).

**Table 2:** Role of MCs and their mediators on the biology of cultured tumor cells lines

Cell line of tumor cells	MC mediator, source, or cell line	Brief description	Ref
KMH2 (human Hodgkin’s lymphoma cell line)	BMMCs of C57BL mice	BMMCs could proliferate KMH2 cells.	[30]
OSRC-2 cells (renal cell carcinoma cell line)	HMC-1	Co-culture with human umbilical vein endothelial cell (HUVEC) showed that HMC-1 released mediators contribute OSRC-2-induced HUVEC recruitment and promote the formation of capillary tubes.	[24]
Thyroid cell lines including Nthy-ori-3-1, TPC-1, NIM-1, BCPAP, 8505c, and CAL62	HMC-1 and LAD2	MC-released IL-8 promotes epithelial–mesenchymal transition (EMT) and stemness of cultured thyroid cancer cells through IL-8–Akt–Slug pathway.	[31]
Membranes derived from A549, H1299, SK-LMS-1, and Panc-1	HMC-1	Culturing HMC-1 with membrane fragments of tumor cells could promote phosphorylation of the MAP kinases ERK1/2 in MCs and activated them.	[32]
Lung carcinoma cell lines A549 and H520	MC chymase	Dose-dependent chymase decreased the rate of proliferation of both cell lines after 24 h post treating. It also hampered the A549 cells adhesion ability, downregulated the expression of E-cadherin	[33]
Glioma cell lines U2987MG and U3086MG	LAD2 cells	Conditioned medium obtained from human glioma cells could induce MC activation and release of IL-6, IL-8, VEGF, and TNF-α. “Tumor educated” MCs could reduce the ability of glioma cells to proliferate and migrate and self-renewal capacity through inactivation of the STAT3 signaling pathway.	[34]
Colon cancer cells HT29 and Caco2	Primary human MCs generated from CD34+ peripheral stem cells in the presence of IL-3 and SCF	In transwell migration assay, the colon cancer cells HT29 and Caco2 could recruit MCs by releasing CCL15 or SCF, respectively. MCs supported the proliferation of colon cancer cells by releasing protumorigenic mediators.	[29]

**Table 3:** MC mediators in the modeling of the tumor microenvironment

Mediator	Involved stage(s)	Comments	Ref
Chymase	Angiogenesis Development of tumor	Induces the proliferation of endothelial cells, induces in vitro vascular tube formation, and degrades the matrix of connective tissue to provide space for neovascular development	[24]
Tryptase	Angiogenesis Tumor cell proliferation Metastasis	Nonclassical proangiogenic mediator Acts in a paracrine manner Tryptase degrades ECM components and activates MMPs Stimulates the proliferation of endothelial cells and promotes the activation of plasminogen activator	[35] [35] [36] [37]
VEGF	Angiogenesis	Acts as a classical proangiogenic factor	[35]
Histamine	Tumor cell proliferation Angiogenesis	Promotes the proliferation of tumor cells Promotes angiogenesis by acting on both H1 and H2 receptors	[10] [38]
TNF-α	Promoting inflammation	Recruits other immune cells including neutrophils to the tumor site	[39]
MMP9	Development of tumor	Promotes tumor invasiveness, mobilizes VEGF from ECM, and supports neoangiogenesis Capable of degrading fibronectin and type IV, V, VII, and X collagens	[40, 41] [38]
MMP-2	Tissue remodeling Development of tumor	Promotes tissue remodeling during neovascularization Capable of degrading fibronectin and type IV, V, VII, and X collagens	[24] [38]
Tissue inhibitors of metalloproteinases (TIMPs)	Tissue remodeling	Promote tissue remodeling during neovascularization	[24]
Nerve growth factor (NGF)	Angiogenesis Development of tumor	Promotes angiogenesis in vivo Induces proliferation of ECs in vitro	[38]
S1P	Development of tumor	S1P activates NF-κB and links inflammation with cancer Contributes to the accumulation of Tregs and tumor development Regulates the activity and expression of HIF1α, the main regulator of hypoxia in tumor	[42]

## Tumor Microenvironment

### Cells of the Adaptive Immune System in TME

To generate an effective antitumor response by T cells, they need to be activated by tumor-associated antigens (TAA) presented by dendritic cells (DCs) that reside in peripheral lymph nodes (LNs). CD8+ T cells activated after recognizing TAA presented by MHC class I molecules on cancer cells can eliminate tumor cells via the action of perforin–granzyme or the Fas ligand (FasL)/TRAIL pathway [43]. Activated Th1 cells release IFNγ, which is a well-known antitumor cytokine that activates macrophage, promotes antigen processing and presentation by APCs, and inhibits angiogenesis [44]. Additionally, Th1 cells along with cytotoxic T lymphocytes (CTLs) produce IFNγ to induce the ability of monocytes and macrophages to produce CXCL9 and CXCL10, which act as angiostatic factors [3]. IFNγ is capable of inhibiting tumor angiogenesis by hampering the proliferation of ECs [3]. Tumor-infiltrating Th2 cells secrete IL-4, which supports the differentiation of tumor-infiltrating monocytes and macrophages into M2-like TAMs (tumor-associated macrophages) [3]. Th2 cells also induce the production of IgG by B cells, which promotes the production of angiogenic factors by FcγR activation on macrophages via [3].

### Cells of Innate Immune System in TME

**Fig. 2:**
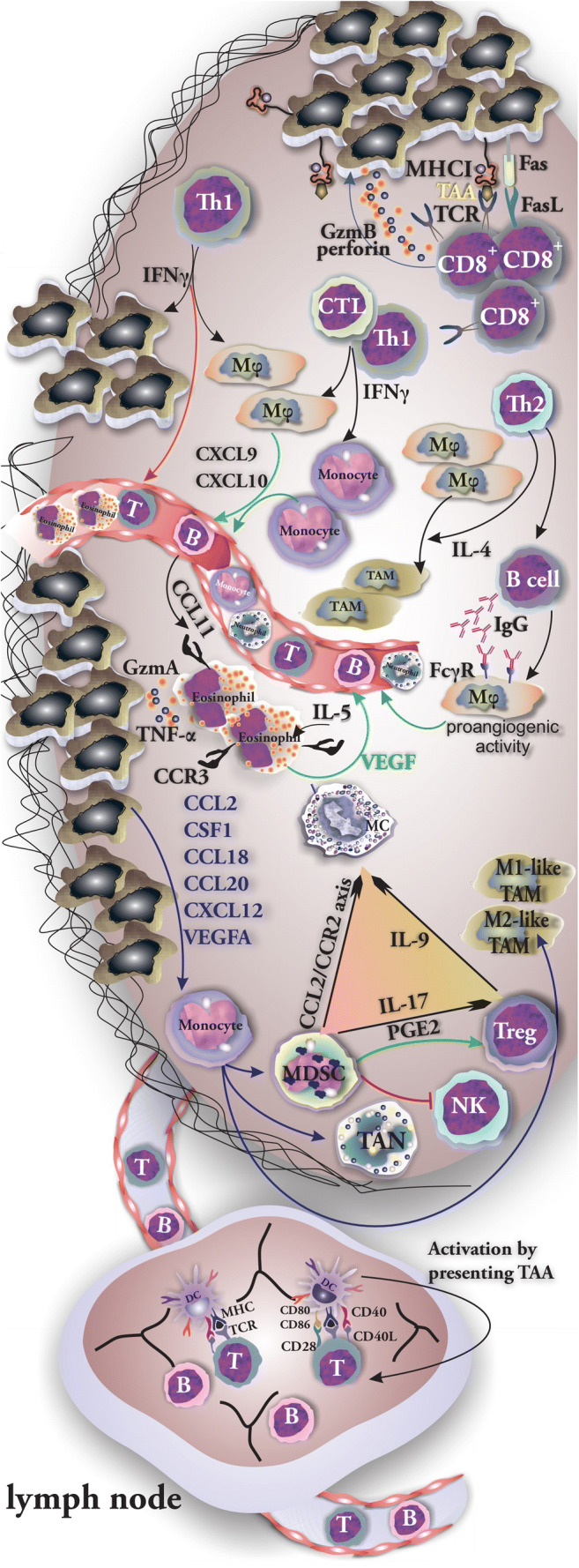
Cells of innate and specific immune system are involved in tumor biology. The role of tumor-residing or recruited cells of both innate and specific immune system in progression and suppression of tumor growth. The positive loop among tumor MCs, Tregs, and MDSCs has been shown. CD8+ recognizes tumor cells and eliminates them by releasing granzyme and perforin. Recruited monocytes give rise to other cell types mainly MDSC, TAM, and TAN. MCs induce the mobilization and infiltration of MDSCs to tumor through CCL2/CCR2 axis. MDSCs attract Treg cells and support their immunosuppressive activity by production of IL-17. Tregs in return produce IL-9 which acts as a survival factor of MCs

The presence of natural killer cells (NK cells) and natural killer T cells in the majority of solid tumors often means a good prognosis [1]. Within the TME, NK cells and CTLs are capable of recognizing malignant cells owing to the expression of NKG2D and T cell receptors, respectively [45]. Monocytes are recruited from the circulation and differentiate into macrophages within the TME where stromal and tumor cells provide chemokines and growth factors mainly CCL2, CSF1, CCL18, CCL20, CXCL12, and VEGF-A [46]. After being recruited to the hypoxic TME, monocytes give rise to other cell types mainly MDSC, TAM, and tumor-associated neutrophils (TANs) and induce a differential and functional immature phenotype of DCs [43]. DCs participate in antitumor immune responses by cross-presentation of antigens and generation of antitumor CTLs [44]. Tumor-associated macrophages (TAMs) may have a protumoral role by promoting the processes of angiogenesis and metastasis and hampering T cell–dependent antitumor responses [46]. There are two determined phenotypes of TAMs: M1-like TAMs orchestrate immune response and normalize abnormal tumor vascular system through which they make chemotherapy agent accessible to tumor cells and contribute to the regression of tumor growth. Unlike them, M2-like TAMs promote immunosuppression and support the formation of abnormal vessels in TME that lead to tumor progression [47]. Cytokines released from tumor-residing cells including tumor cell–derived IL-4, IL-10, CCL2, CCL3, CCL4, and CSF1; Treg-derived IL-10; B cell–produced immunoglobulins; Th2-derived IL-4 and IL-13; and MSC-derived MFG-E8 promote the polarization into a protumor phenotype. Additionally, TAMs residing in the TME can produce MIF, IL-10, and CXCL12 which may further promote the polarization [46]. Interestingly, hypoxic cells inside the tumor—in return—release a variety of cytokines including sphingosine-1-phosphate (S1P), IL-6, eotaxin, and oncostatin M through which they induce M2 macrophage/TAM polarization [42]. Myeloid-derived suppressor cells (MDSCs) are commonly found in different types of cancers and within the TME. They are capable of suppressing NK and T cells through different mechanisms including direct cell-to-cell interactions and through cytokine release [48]. MDSC-released PGE2 promotes the development of Tregs, induces the production of immunosuppressive chemokines, and promotes barrier function of ECs through the inhibition of transendothelial T cell migration [43]. Interestingly, MCs were reported to mobilize and promote the infiltration of MDSCs via the CCL2/CCR2 axis in TME where they produce IL-17 through which Tregs accumulate onsite. Treg-released IL-9 completes this positive loop by supporting MC survival [49]. Neutrophils can be recruited to the TME by MC-derived chemokines including CCL1, CCL2, CCL3, CCL4, CCL5, and CXCL8 [50]. Neutrophils secrete VEGF-A, FGF2, and CXCL8 that promote angiogenesis [3]. Under the influence of CCL11 (eotaxin-1) that binds to CCR3, eosinophils are recruited to the TME. Although eosinophils possess tumoricidal activity by releasing granzyme A and TNF-α, after activation by IL-5, they also release VEGF and can promote angiogenesis [51]. In addition to immune cells, tumor-residing fibroblasts and stromal cells act as a source of cytokines mainly hepatocyte growth factor (HGF), fibroblast growth factor (FGFs), and CXCL12 that are capable of supporting the growth and survival of malignant cells and promoting the infiltration of variety of cells [1] (Fig. 2).

## Mast Cell Recruitment to Tumor

**Fig. 3:**
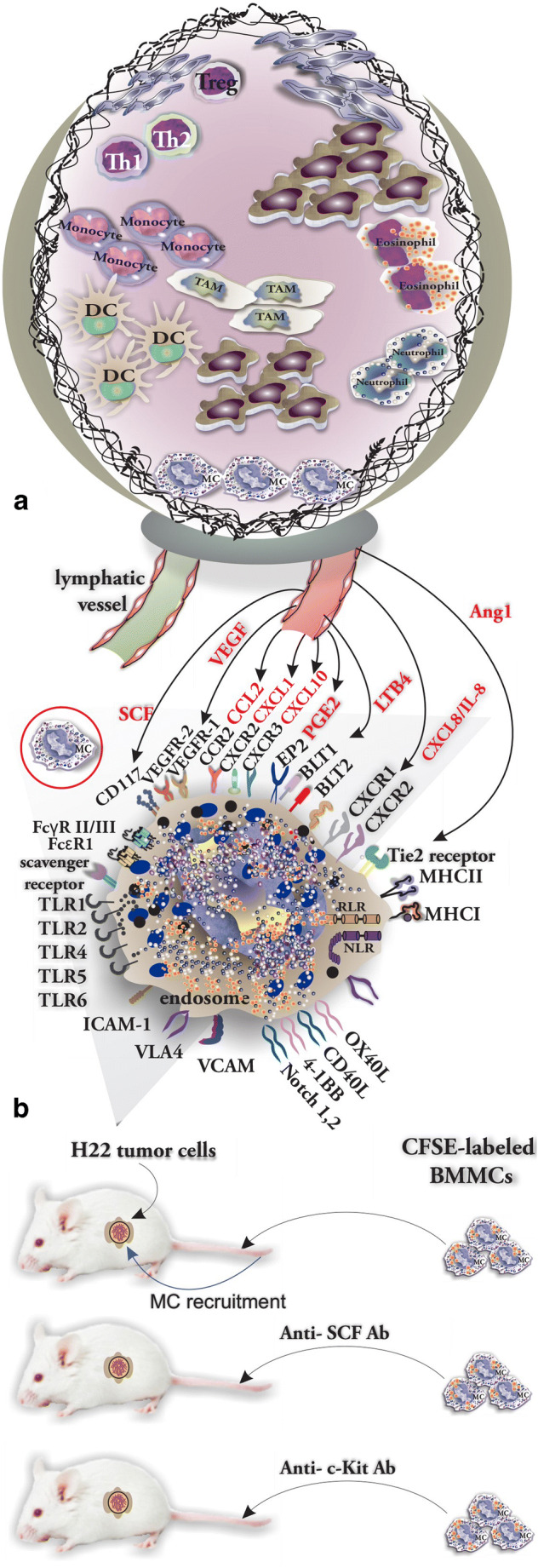
The recruitment of MCs in tumor is organized by a complicated ligand-receptor network. **a** Chemokine network involved in MC recruitment to TME (chemokines are shown in red). **b** The protocol used by Huang to determine the tumor-released SCF as the main chemokine involved in MC recruitment

MCs have a strictly regulated trafficking to the TME owing to interactions of locally produced chemokines and receptors expressed on MCs. One of the main chemoattractant factors produced by tumor cells is SCF, which is also the main survival factor for MCs. Furthermore, a variety of other chemokine/receptor interactions such as LTB4 with BLT1 and BLT2 [52], PGE2 with the EP2 receptor, VEGF via VEGFR-1 and VEGFR-2, angiopoietin 1 (Ang1) which acts on Tie2 receptor, and also CXCL8/IL-8 interactions with CXCR1 and CXCR2 play a crucial role in the attraction of MCs to the sites of chronic inflammation including TME. Localization of MCs in the TME is determined by interactions of CCR2, CXCR2, and CXCR3 with their respective ligands CCL2, CXCL1, and CXCL10 [53]. Roy et al. [54] investigated the recruitment pattern of MCs to glioma tumors and reported that glioma-derived plasminogen activator inhibitor-1 (PAI-1) promotes MC recruitment and that the level of PAI-1 correlates with the rate of MC recruitment. Additionally, macrophage migration inhibitory factor (MIF) released by glioma cells contributes to the recruitment of MCs by inducing phosphorylation of STAT5 [55]. Also, glioma cells release CXCL12 that acts as MC chemotaxin by engaging CXCR4 [56] (Fig. 3a). Huang et al. [2] showed that both anti-SCF and anti-c-Kit antibodies suppressed the infiltration of injected bone marrow–cultured mast cells into inoculated H22 tumors in mice (Fig. 3b).

## Role of MCs in Angiogenesis

The tumor vasculature system does not support the growing mass of cells with adequate blood flow. Therefore, tumor cells face an imparted supply of nutrients, gas exchange, and drainage of tumor-produced metabolites. This situation consequently leads to the creation of a hypoxic and acidotic TME through which angiogenesis and the formation of heterogeneous new vessels are induced to compensate the shortage of blood supply [1, 47, 57]. Additionally, endothelial cells (ECs) that form the tumor vessels are capable of suppressing the recruitment, adhesion, and activity of circulating T cells by acting as physical barriers to immune cells [43]. There is a number of angiogenic mediators released by MCs in the tumor microenvironment including IL-8, NGF, TNF-α, TGF-β, and urokinase-type plasminogen activator (PA) [3]. MC tryptase promotes the proliferation of endothelial cells [26], facilitates the in vitro vascular tube formation, and degrades the matrix of connective tissue to create adequate space for neovascular development [3]. Additionally, histamine acting on H1R and H2R stimulates the formation of new vessels [3]. In addition to playing a role in angiogenesis via VEGF-A and VEGF-B [58], MCs are also involved in lymphangiogenesis by releasing VEGF-C and VEGF-D [59]. Blair et al. investigated in a co-culture model of the human MC cell line (HMC-1) and dermal microvascular endothelial cells (HDMEC) the impact of MC mediators on tube formation. Calcium ionophore–activated MCs were found to promote the formation of tubes. Tryptase was shown as the main mediator involved in neovascularization and endothelial cell proliferation, and tube formation was suppressed by tryptase inhibitors, recombinant leech–derived tryptase inhibitor, and bis(5-amidino-2-benzimidazo-lyl) methane [60]. Guo et al. assessed in a similar approach the effects of tryptase released by human recombinant lung MC on the proliferation and tube formation ability of human umbilical vein endothelial cells (HUVEC). It was shown that the incubation of the cells with tryptase significantly increased their viability and proliferation. Additionally, treating the cells with nafamostat, a tryptase inhibitor, reversed this effect. PD98059, an inhibitor of ERK phosphorylation, suppressed the promoting effects of tryptase. Moreover, MC tryptase did not only induce the proliferation of HUVECs, but it even promoted the tube formation [26]. In addition, it was shown that MC tryptase induced the tumorigenesis and angiogenesis in vivo after inoculation of PANC-1 (pancreatic cancer cell line) into nude mice. Tumor cells injected together with tryptase were larger than those developed in non-treated mice and nafamostat was able to suppress these tumorigenetic effects of tryptase [26].

## Evidence of MC Involvement in Tumor Biology: Lessons from Mouse Models

Studies revealing possible mechanisms by which MCs support tumorigenesis or suppress tumor growth largely used mouse strains deficient for a specific receptor or receptor ligand. Specifically, reconstitution of MC-deficient mice with MCs obtained from WT mice or knock-out mice demonstrated the crucial role of specific receptor/ligand in such a model on the progression of tumor growth. He et al. investigated the effects of MC deficiency on the development of mammary tumors through crossing the Kit^W-sh/W-sh^ with the mammary tumor transgenic mouse (Tg) strain MMTV-polyomavirus middle T antigen best known as PyMT model. Female Kit^+/W-sh^ and male PyMT/Kit^+/W-sh^ mice were used to generate female PyMT/wild-type (WT) and PyMT/Kit^W-sh/W-sh^ littermates. They found that tumor progression and further metastasis were significantly reduced in PyMT/Kit^W-sh/W-sh^ mice when compared with PyMT/wild-type mice (WT) [61]. In a similar protocol, Bodduluri et al. [62] mated ACKR2^−/−^ mice with Apc^Min/+^ mice (determined by having a mutation in the adenomatous polyposis coli gene) to generate ACKR2^−/−^Apc^Min/+^. Atypical chemokine receptor 2 (ACKR2) is a decoy receptor that binds to and internalizes inflammatory chemokines. Generated ACKR2^−/−^Apc^Min/+^ mice were found to develop tumors with infiltrated MCs. ACKR2^−/−^BLT1^−/−^Apc^Min/+^ mice, which lack the LTB4 receptor, showed impaired CD8+ recruitment into tumors, and this made them highly susceptible to develop intestinal tumors. These studies indicated that LTB4 produced by MC may support CTL recruitment to TME and antitumor responses [62]. Melillo et al. [27] showed that MCs are able to enhance the growth of human thyroid cancer cells in athymic nu/nu mice. Co-injection of 8505-C cells and HMC-1 cells resulted in earlier tumor formation with a higher tumor volume when compared with tumors formed after injection of 8505-C cells alone.

## MC Interactions with Tumor Cells

**Fig. 4:**
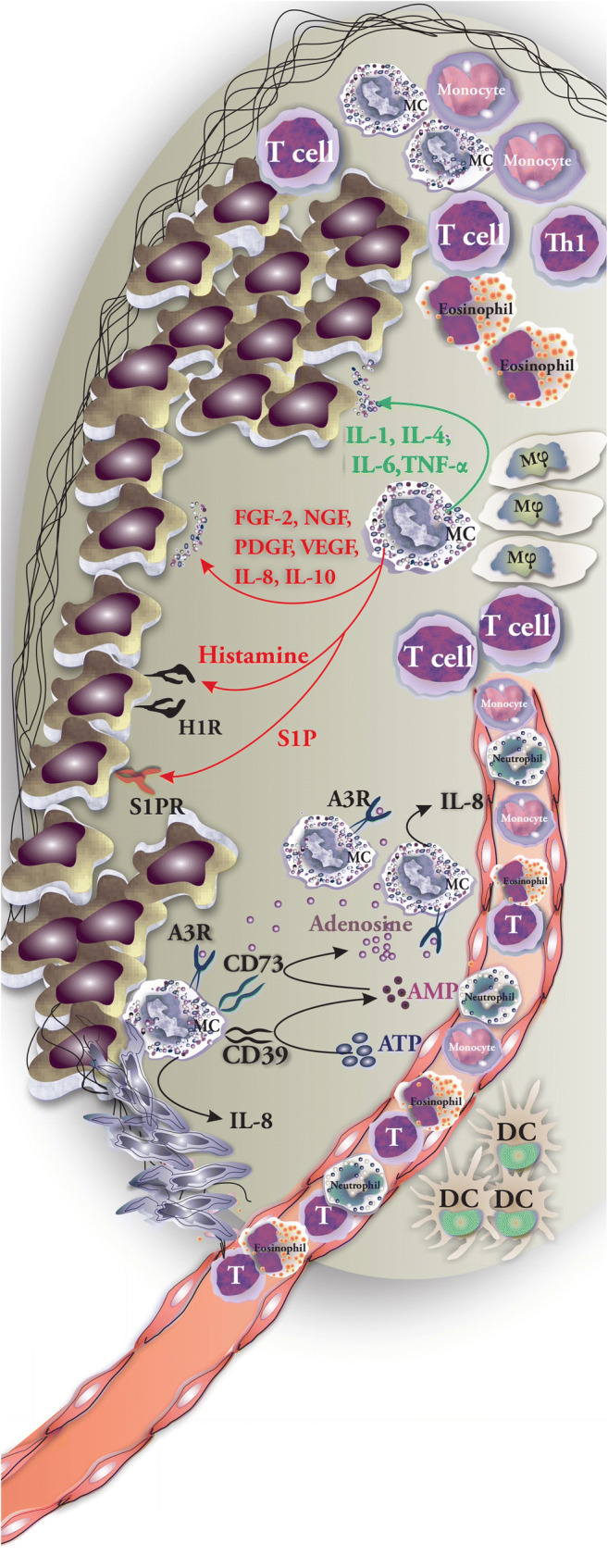
Possible interactions of MCs and tumor cells and cell-to-cell interaction with other immune cells. Possible interactions between MCs and tumor cells include releasing mediators (tumor growth–supporting MC mediators are shown in red and tumor-suppressing MC mediators are listed in green) and direct cell-to-cell contacts that result in activation or suppression of cells

MCs through releasing IL-1, IL-4, IL-6, and TNF-α can actively participate in the elimination of tumor cells and rejection of tumors [63]. Conversely, mediators released by MC such as FGF-2, NGF, PDGF, VEGF, IL-8, and IL-10 promote the expansion of tumor cells [36]. Additionally, histamine induces tumor cell proliferation by acting on tumor surface expressed H1 receptors (H1R) [36]. In the TME, MCs are main contributors to S1P production along with tumor cells. S1P promotes proliferation, migration, and survival of tumor cells [64]. In solid tumors such as thyroid tumors, histamine engagement of H1R and H2R results in tumor cell proliferation. Moreover, CXCL1/GRO-α and CXCL10/IP-10 have been reported to support invasion, proliferation, and survival of tumor cells by acting on CXCR2 and CXCR3, respectively [58]. Cell-to-cell interactions between MCs and tumor cells may result in MC activation and release of mediators. Such interactions induce the formation of adenosine in an autocrine manner by MCs via a CD73-dependent mechanism. Adenosine then engages the adenosine A3R through which ERK1/2 MAP kinases are activated and IL-8 is produced and released by MCs into the TME [32] (Fig. 4). Chen et al. investigated the role of MCs in the progression of renal cell carcinoma (RCC) and the possible mutual interaction of MCs and tumor cells. First, they added conditioned medium (CM) from RCC OSRC-2 cells or from OSRC-2 plus HMC-1 into the lower chamber of a Transwell system, while human umbilical vein endothelial cells (HUVECs) were placed in the upper chamber. With this setup, they showed that HMC-1 CM promoted the OSRC-2-induced HUVEC recruitment. Using bevacizumab or cromolyn to inhibit MC degranulation suppressed HUVEC recruitment and the formation of capillary tubes in vitro. To determine the ability of MCs in enhancing the angiogenesis, OSRC-2 cells and HMC-1 cells were injected subcutaneously into the dorsal region of nude mice. Co-injection of HMC-1 and OSRC-2 promotes the formation of microvessels as compared with the injection of OSRC-2 alone [24]. It was suggested that PI3K → AKT → GSK3β (a downstream substrate of PI3K/Akt pathway) signaling pathway induces the expression of adrenomedullin (AM) through which MCs are recruited to TME, where they recruit endothelial cells by releasing VEGF and FGF-2, induce tissue remodeling by secreting tryptase and MMPs, and consequently promote angiogenesis in RCC [24].

## MC and Extracellular Matrix During Tumor Development

ECM regulates a variety of biological functions of both normal and tumor cells including cellular migration and adhesion [65]. In return, the resident tumor cells, fibroblasts, endothelial cells, recruited inflammatory cells, and pericytes secrete ECM proteins [65]. MC-derived tryptase may promote neovascularization by activating MMPs. These enzymes play a key role in degrading the ECM and discharging angiogenic factors [66]. Tryptase may activate the plasminogen activator and induce the release of VEGF and FGF-2 from their extracellular matrix-bound state [37]. Maniga et al. reported the accumulation of MCs in tumors of breast cancer and that MC-derived tryptase is capable of initiating fibroblast differentiation and promoting stromal remodeling [17]. MMP-9 is one of the main MMPs capable of degrading and remodeling ECM leading to an alteration of the cellular microenvironment. Under the influence of MC chymase, E-cadherin molecules that connect epithelial cells at adherent junctions are cleaved and their expression is decreased. It is also reported that chymase increases the expression of MMP-9 in tumor cells. Chymase therefore promotes the separation, proliferation, and relocation of cell clusters through acting directly and indirectly on ECM to support metastasis [33].

## MCs as Therapeutic Targets

The relationship between MC density of tumors, the progression of angiogenesis, and tumor development may highlight the possible role of MCs in tumor biology. Therefore, the possibility of targeting MC activation [67, 68], inhibiting the release of mediators using c-Kit receptor tyrosine kinase inhibitors (TKI) (including imatinib, masitinib [69]), or using tryptase inhibitors (mainly gabexate mesylate and nafamostat mesylate, both inhibitors of trypsin-like serine proteases [69, 70]) may be valuable therapeutic approaches to control the tumor development [71]. Masitinib, a TKI that targets c-kit receptors (CD117), has been used in veterinary medicine for years, and lately, human clinical trials were initiated to test its clinical efficacy as single or add-on treatment human cancers such as mastocytosis, gastrointestinal stromal tumors (NCT00998751), colon cancer (NCT03556956), prostate cancer (NCT03761225), and pancreatic cancer (NCT03766295) [72]. Imatinib due to its property of inhibiting the protein tyrosine kinase BCR/ABL is used in the treatment of chronic myeloid leukemia (CML) [72]. Additionally, silymarin inhibits the recruitment of MCs and reduces the expression of MMP-2 and MMP-9 [72]. It is generally believed that inflammation promotes tumor growth and accelerates metastasis and the process of angiogenesis. MCs are an important source of proinflammatory cytokines in TME. Agents that hamper their ability to produce proinflammatory cytokines may be of therapeutic importance in controlling the tumor growth. Most recently, Nam et al. [73] reported that Dp44mT is able of blocking caspase-1 and NF-κB and consequently mitigate the production of IL-1β, IL-6, TNF-α, and thymic stromal lymphopoietin (TSLP) by MC. VEGF-centered anti-angiogenic tumor therapy could fail due to resistance. It has been reported that upon therapy, MCs release granzyme B, which mobilizes proangiogenic factors from the tumor matrix mainly laminin- and vitronectin-bound FGF-1 and GM-CSF. Wroblewski et al. showed that MCs release FGF-2 that acts on ECs and induces their proliferation and promotes angiogenesis. The combination of cromolyn to prevent MC degranulation along with anti-angiogenic therapy promoted the therapeutic efficacy [74]. The engagement of TLR2 on MC has shown to stimulate tumor growth, and blocking this pathway may be promising in designing of immunotherapeutic strategies. In a 3D co-culture setup, FSL-1-mediated TLR2 stimulation of MCs supported the growth of colon cancer spheroids [29]. On the other hand, it was shown in an orthotopic B16.F10 melanoma model that TLR2-activated MC could also inhibit tumor growth by an IL-6-dependent mechanism [75].

## Parallels with Autoimmunity

The role of MCs in tumor development draws parallels with their projected role in autoimmunity and chronic inflammatory diseases. The chance of initiation of autoimmune disease depends largely on the disruption of the balance between the pro- and anti-inflammatory cell populations and their released cytokines. MCs not only release a wide range of proinflammatory mediators but also are considered as the cells producing immunosuppressive cytokines. One possible pathway is the ability of MCs in the production of IL-10 thus supporting the increase in the number of Tregs in the draining lymph nodes. Tregs counteract the proinflammatory Th1 and Th17 cells [76]. The role of MCs in the pathology of a variety of autoimmune diseases including rheumatoid arthritis (RA), multiple sclerosis (MS), type I diabetes mellitus (T1DM), and systemic lupus erythematosus has been investigated. MC-derived TNF, IL-1*β*, IL-17, and tryptase have been reported to play a role in the pathogenesis of RA. Tryptase for instance, through acting on PAR2, activates synovial fibroblasts to express more proteases that degrade cartilage and bone [77]. Furthermore, MCs in response to anti-citrullinated protein antibodies (ACPA) and TLR ligands become activated and release IL-8, TNF-α, and LTs which act as neutrophil chemoattractants to synovial fluid which results in the aggravation of inflammation [78]. In MS, autoreactive T cells after becoming activated in the periphery infiltrate the CNS and act as effector cells in the pathology of the disease. The detrimental role of MCs in MS includes (1) supporting the recruitment of autoreactive T cells by releasing CCL2, CCL3, CCL4, CCL5, and IL-16 [79], (2) activating and promoting the differentiation of Th1, Th2, and Th17 subsets by releasing IL-4, IL-6, IL-10, IL-13, TGF-β, and TNF-α [8], (3) increasing brain–blood barrier (BBB) permeability by releasing histamine [8], and (4) degrading myelin by releasing proteases [80]. The initiation and development of type 1 diabetes (T1DM) depends on the autoimmune destruction of pancreatic β cells. Interestingly, individuals with T1D have higher levels of circulating IgE when compared with healthy individuals [81]. Additionally, the number of MCs increases in pancreatic lymph nodes. These cells show an overrepresentation of mediators including IL-5, protease 1, trypsinogen, carboxypeptidase A, and phospholipase Cγ [82]. Activation pathways and MC mediators involved in autoimmunity certainly have similarities with those found in tumor biology, although the specific microenvironment will ultimately determine the production and role of specific MC mediators in the pathophysiology of the disease.

## Conclusion and Discussion

The nature of the interaction between tumor cells and TME-resident cells is mutual in which the behavior of tumor cells determines the fate of tumor and influences the biology of cells of TME, and conversely, the TME-resident cells may affect the way a tumor initiates, grows locally, or spreads throughout the body [48]. The interaction of MCs with other cell types in TME should be extensively investigated to clarify other possible interactions and potential prognostic significance. In this regard, Leni et al. [39] studied MC–neutrophil interactions within heterotypic aggregations in TME of patients with gastric carcinoma and reported that MCs are able to release their mediators in small amounts through a mechanism called kiss-and-run fusion. Most recently, researchers benefitted from a novel computer-aided tissue analysis method for identifying and counting MCs in TME for the purpose of eliminating the operator bias. Using this method, Eder et al. [20] reported the infiltration of MCs in different zones of cutaneous T cell lymphomas. Shikotra et al. focused on the cytotoxic activity of MCs and their ability to express TNFα and reported that the presence of TNFα releasing MCs in non-small cell lung cancer tumors may be related to extended survival of patients. Promoting the cytotoxic activity of MCs seems to be a possible approach to control some tumors which needs to be further investigated [13]. Focusing on the mechanisms of MC activation in tumors and releasing inflammatory cytokines could provide novel tumor controlling strategies. Of the mechanisms described for MC activation, free light chains (FLC) have been investigated in several models. The inhibition of FLC-mediated MC activation was reported in a murine B16F10 melanoma model to reduce the tumor growth [83]. In other studies, the spatial distribution of MCs around vessels and glands in gastric carcinoma(GC) was investigated using IHC and computer-assisted analysis of tissue specimen and it was concluded that in GC grade II, there is a spatial association of chymase+ MCs showing that MCs were located at a shorter distance from the vessels [84]. Investigations aimed to reveal the architecture and spatial distribution of may give more detailed information regarding role of MCs in tumor biology. Assessing MC heterogeneity in benign and malignant solid tumors may be helpful for targeting them and avoid further MC-orchestrated tumor angiogenesis. In this regard, Globa et al. reported phenotype heterogeneity of MCs in prostate cancer. They concluded that tryptase+/CD117+/chymase− and tryptase−/chymase+/CD117+ phenotypes were located in peritumoral areas of patients with benign lesions, while tryptase+/chymase+/CD117+ MCs were frequently found in malignant lesions [85]. Most recently, Molderings et al. analyzed German and American individuals with systemic MC activation syndrome (MCAS) and reported a higher chance of developing solid tumors especially melanoma, breast cancer, thyroid, ovary, lung, and cervix uteri. According to the high tissue burden of infiltrating MCs in these patients, they may need further investigations to reveal the prevalence of solid tumors in MCAS patients of other ethnic backgrounds and the mechanism by which they become more susceptible to develop solid tumors [86]. In addition, special effort is needed to determine the MC mast cell make-up in the tumor environment, because interaction with the complex tumor environment may alter the functional expression of various membrane receptors. In this regard, Yu et al. [87] reported the increased expression of Siglec-6 (a sialic acids binding receptor) on MC in vitro models for human colon cancer. Upon co-incubation with colon cancer cells or hypoxia, Siglec-6 is upregulated on MCs and reduces MC activation [87]. Based on our current knowledge about the involvement of MCs in inducing inflammation and angiogenesis in the TME, they may be promising targets in the adjuvant treatment of cancers by—on the one hand—selective inhibition of angiogenesis and tissue remodeling, targeting the release of tumor-promoting molecules, and targeting MC-orchestrated immune-suppression, and—on the other hand—stimulating their ability to produce cytotoxic cytokines resulting in an enhanced tumor degradation [63].

## Abbreviations

BMMCs: Bone marrow–derived MCs
TME: Tumor microenvironment
TAMs: Tumor-associated macrophages
MFG-E8: Mesenchymal stromal cell–derived milk fat globule-epithelial growth factor 8 protein
MDSC: Myeloid-derived suppressor cells
TRAIL: TNF-related apoptosis-inducing ligand
NGF: Nerve growth factor
PDGF: Platelet-derived growth factor
LN: Lymph node
